# Supplementary material for: Determinants of breastfeeding attitudes of mothers in Jordan: A cross-sectional study
Source: PLoS One. 2023 May 5;18(5):e0285436. doi: 10.1371/journal.pone.0285436 (PMC10162564; doi:10.1371/journal.pone.0285436)
Supplement: Attachment — Submitted filename: PONE-D-22-35675_reviewer--OMR.pdf [file pone.0285436.s003.pdf]

PLOS ONE

Determinants of Breastfeeding Attitudes of Mothers in Jordan: A Cross-sectional Study

--Manuscript Draft--

**Manuscript Number:**	PONE-D-22-35675
**Article Type:**	Research Article
**Full Title:**	Determinants of Breastfeeding Attitudes of Mothers in Jordan: A Cross-sectional Study
**Short Title:**	Determinants of Breastfeeding Attitudes of Mothers in Jordan
**Corresponding Author:**	Sireen Alkhaldi, DrPH<br>The University of Jordan School of Medicine<br>Amman, JORDAN
**Keywords:**	Determinants; breastfeeding; attitude; Jordan; IIFAS; mothers; Iowa Infant Feeding Attitude Scale
**Abstract:**	Breastfeeding provides the best nutrition ever for an infant. Therefore, it is an infant's human right. However, breastfeeding practice is on decline globally. Attitude toward breastfeeding may determines the practice. This study aimed to examine postnatal mothers' attitudes to breastfeeding and its determinants. A cross-sectional study was conducted, and data on attitude were collected using the Iowa Infant Feeding Attitude Scale (IIFAS). Three hundred and one (301) postnatal women were recruited from a major referral hospital in Jordan. Data on sociodemographic characteristics, pregnancy and delivery outcomes were collected. SPSS was used to analyze the data and t-test, ANOVA and multiple linear regression were performed to identify the determinants of attitudes to breastfeeding. The mean attitude score for participants was 65.0 ±7.15, indicating a neutral attitude. Factors associated with positive breastfeeding attitude were high income (p=0.048), pregnancy complications (p= 0.049), delivery complications (p= 0.008), prematurity (p= 0.042), intention to breastfeed (p= 0.002) and willingness to breastfeed (p= 0.005). Multiple linear regression results indicated higher income and intention to breastfeed as predictors of positive breastfeeding attitude (AOR 0.019; 95% CI 0.30-2.91 and AOR 1.52; 95%CI 0.05-10.20 respectively). Mothers in Jordan have neutral attitude to breastfeeding. Breastfeeding promotion programs and initiatives should target low-income mothers and the general population. Policymakers and health care professionals can use the results of this study to improve breastfeeding rate in Jordan.
**Order of Authors:**	Sireen Alkhaldi, DrPH
	Oqba Al-Kuran
	Mai M. AlAdwan
	Tala A. Dabbah
	Heyam F. Dalky
	Eiman Badran
**Additional Information:**	
**Question**	**Response**
**Financial Disclosure**<br><br>Enter a financial disclosure statement that describes the sources of funding for the work included in this submission. Review the submission guidelines for detailed requirements. View published research articles from *PLOS ONE* for specific examples.	The authors received no specific funding for this work

Powered by Editorial Manager® and ProduXion Manager® from Aries Systems Corporation

This statement is required for submission and **will appear in the published article** if the submission is accepted. Please make sure it is accurate.<br><br>**Unfunded studies**<br>Enter: *The author(s) received no specific funding for this work.*<br><br>**Funded studies**<br>Enter a statement with the following details:<br>• Initials of the authors who received each award<br>• Grant numbers awarded to each author<br>• The full name of each funder<br>• URL of each funder website<br>• Did the sponsors or funders play any role in the study design, data collection and analysis, decision to publish, or preparation of the manuscript?<br>• **NO** - Include this sentence at the end of your statement: *The funders had no role in study design, data collection and analysis, decision to publish, or preparation of the manuscript.*<br>• **YES** - Specify the role(s) played.<br><br>* typeset	
**Competing Interests**<br><br>Use the instructions below to enter a competing interest statement for this submission. On behalf of all authors, disclose any competing interests that could be perceived to bias this work—acknowledging all financial support and any other relevant financial or non-financial competing interests.<br><br>This statement is required for submission and **will appear in the published article** if the submission is accepted. Please make sure it is accurate and that any funding sources listed in your Funding Information later in the submission form are also declared in your Financial Disclosure statement.<br><br>View published research articles from *PLOS ONE* for specific examples.	The authors have declared that no competing interests exist

**NO authors have competing interests**<br><br>Enter: *The authors have declared that no competing interests exist.*<br><br>**Authors with competing interests**<br><br>Enter competing interest details beginning with this statement:<br><br>*I have read the journal's policy and the authors of this manuscript have the following competing interests: [insert competing interests here]*<br><br>* typeset	
**Ethics Statement**<br><br>Enter an ethics statement for this submission. This statement is required if the study involved:<br><br>• Human participants<br>• Human specimens or tissue<br>• Vertebrate animals or cephalopods<br>• Vertebrate embryos or tissues<br>• Field research<br><br>Write "N/A" if the submission does not require an ethics statement.<br><br>General guidance is provided below. Consult the submission guidelines for detailed instructions. **Make sure that all information entered here is included in the Methods section of the manuscript.**	The ethical committee (IRB) in the University of Jordan Hospital approved this study (no. 2019-229).

Powered by Editorial Manager® and ProduXion Manager® from Aries Systems Corporation

**Format for specific study types**<br><br>**Human Subject Research (involving human participants and/or tissue)**<br>• Give the name of the institutional review board or ethics committee that approved the study<br>• Include the approval number and/or a statement indicating approval of this research<br>• Indicate the form of consent obtained (written/oral) or the reason that consent was not obtained (e.g. the data were analyzed anonymously)<br><br>**Animal Research (involving vertebrate animals, embryos or tissues)**<br>• Provide the name of the Institutional Animal Care and Use Committee (IACUC) or other relevant ethics board that reviewed the study protocol, and indicate whether they approved this research or granted a formal waiver of ethical approval<br>• Include an approval number if one was obtained<br>• If the study involved *non-human primates*, add *additional details* about animal welfare and steps taken to ameliorate suffering<br>• If anesthesia, euthanasia, or any kind of animal sacrifice is part of the study, include briefly which substances and/or methods were applied<br><br>**Field Research**<br><br>Include the following details if this study involves the collection of plant, animal, or other materials from a natural setting:<br>• Field permit number<br>• Name of the institution or relevant body that granted permission	
**Data Availability**<br><br>Authors are required to make all data underlying the findings described fully available, without restriction, and from the time of publication. PLOS allows rare exceptions to address legal and ethical concerns. See the PLOS Data Policy and FAQ for detailed information.	No - some restrictions will apply

Powered by Editorial Manager® and ProduXion Manager® from Aries Systems Corporation

A Data Availability Statement describing where the data can be found is required at submission. Your answers to this question constitute the Data Availability Statement and **will be published in the article**, if accepted.<br><br>**Important:** Stating 'data available on request from the author' is not sufficient. If your data are only available upon request, select 'No' for the first question and explain your exceptional situation in the text box.<br><br>Do the authors confirm that all data underlying the findings described in their manuscript are fully available without restriction?	
**Describe where the data may be found in full sentences. If you are copying our sample text, replace any instances of XXX with the appropriate details.**<br><br>• If the data are **held or will be held in a public repository**, include URLs, accession numbers or DOIs. If this information will only be available after acceptance, indicate this by ticking the box below. For example: *All XXX files are available from the XXX database (accession number(s) XXX, XXX.)*.<br>• If the data are all contained **within the manuscript and/or Supporting Information files**, enter the following: *All relevant data are within the manuscript and its Supporting Information files.*<br>• If neither of these applies but you are able to provide **details of access elsewhere**, with or without limitations, please do so. For example:<br><br>*Data cannot be shared publicly because of [XXX]. Data are available from the XXX Institutional Data Access / Ethics Committee (contact via XXX) for researchers who meet the criteria for access to confidential data.*<br><br>*The data underlying the results presented in the study are available from (include the name of the third party*	Data cannot be shared publicly because of privacy issues in the hospital. Data are available from the Corresponding author Institutional Data Access / Ethics Committee (contact via s.alkhaldi@ju.edu.jo) for researchers who meet the criteria for access to confidential data.

and contact information or URL).<br>• This text is appropriate if the data are owned by a third party and authors do not have permission to share the data.<br><br>* typeset	
Additional data availability information:	Tick here if your circumstances are not covered by the questions above and you need the journal’s help to make your data available.

Powered by Editorial Manager® and ProduXion Manager® from Aries Systems Corporation

Manuscript

Click here to access/download;Manuscript;Dr. Sireen Infant Feeding Attitude Manusctipt PLOS ONE.docx

Determinants of Breastfeeding Attitudes of Mothers in Jordan: A Cross-sectional Study

Short title: Determinants of Breastfeeding Attitudes of Mothers in Jordan

Sireen M. Alkhaldi[1*], Oqba Al-Kuran[2], Mai M. AlAdwan[3], Tala A. Dabbah[3], Heyam F. Dalky[4], Eiman Badran[5].

[1] Department of Family and Community Medicine, School of Medicine, the University of Jordan, Amman, Jordan.

[2] Department of Obstetrics and Gynecology, School of Medicine, the University of Jordan, Amman, Jordan.

[3] School of Medicine, the University of Jordan, Amman, Jordan.

[4] Department of Community and Mental Health Nursing, School of Nursing, Jordan University of Science and Technology, Irbid, Jordan.

[5] Department of Pediatrics, School of Medicine, the University of Jordan, Amman, Jordan.

Corresponding Author:

Sireen M. Alkhaldi , DrPH
Department of Family and Community Medicine, School of Medicine,
The University of Jordan, P.O Box: Amman 11942 Jordan. Mobile: + 962 079 6926399.
Email: s.alkhaldi@ju.edu.jo

Determinants of the Breastfeeding Attitudes of Mothers in Jordan: A Cross-sectional Study

Abstract

Breastfeeding provides the best nutrition ever for an infant. Therefore, it is an infant's human right. However, breastfeeding practice is on decline globally. Attitude toward breastfeeding may determines the practice. This study aimed to examine postnatal mothers' attitudes to breastfeeding and its determinants. A cross-sectional study was conducted, and data on attitude were collected using the Iowa Infant Feeding Attitude Scale (IIFAS). Three hundred and one (301) postnatal women were recruited from a major referral hospital in Jordan. Data on sociodemographic characteristics, pregnancy and delivery outcomes were collected. SPSS was used to analyze the data and t-test, ANOVA and multiple linear regression were performed to identify the determinants of attitudes to breastfeeding. The mean attitude score for participants was 65.0 ±7.15, indicating a neutral attitude. Factors associated with positive breastfeeding attitude were high income (p=0.048), pregnancy complications (p= 0.049), delivery complications (p= 0.008), prematurity (p= 0.042), intention to breastfeed (p= 0.002) and willingness to breastfeed (p= 0.005). Multiple linear regression results indicated higher income and intention to breastfeed as predictors of positive breastfeeding attitude (AOR 0.019; 95% CI 0.30-2.91 and AOR 1.52; 95%CI 0.05-10.20 respectively). Mothers in Jordan have neutral attitude to breastfeeding. Breastfeeding promotion programs and initiatives should target low-income mothers and the general population. Policymakers and health care professionals can use the results of this study to improve breastfeeding rate in Jordan.

Keywords: Determinants, breastfeeding, attitude, Jordan, IIFAS, mothers, Iowa Infant Feeding Attitude Scale.

Determinants of the Breastfeeding Attitudes of Mothers in Jordan: A Cross-sectional Study

Introduction:

Breastfeeding enhances bonding between a mother and her infant after birth. It is the most valuable gift a mother can offer to her newborn. The advantages of breastfeeding for both newborns and their mothers are well documented. Increasing the rates of breastfeeding alone can have the potential to save the lives of around a million children under age 5 worldwide [1, 2].

Skin to skin contact and breastfeeding immediately after birth have been reported to minimize infant mortality and promote maternal health [3]. In addition, exclusive breast feeding for six months, continued for two years and beyond with the provision of safe and appropriate complementary foods are the most important strategies for promoting child survival and health [4]. However, the benefits of breastfeeding are underestimated, and the proportion of mothers who breastfeed their newborns is way below the recommended levels globally and Jordan is not an exception [5, 6].

Numerous factors influence both initiation and continuity of breastfeeding. These factors include mothers' age, income, education, mode of delivery and availability of breastfeeding counseling before, during and after delivery, and past breastfeeding experience [7-13]. In addition, health professionals' attitudes, type of health care facility where delivery occurred, and admission of the infant in neonatal intensive care unit (NICU) influence the process of breastfeeding by women [9, 14-17]. Furthermore, several studies have demonstrated that mothers' attitudes toward breastfeeding is a significant predictor of their intention, initiation, and

continuation of [13, 18-23]. Researchers in Jordan demonstrated that a mother's positive attitude toward breastfeeding is a predictor of exclusive breastfeeding. [20, 24].

What determines women’s attitude towards breastfeeding? A large body of research have indicated various factors that influence women’s attitude to breastfeeding, including age, education, family income, employment and previous experience with breastfeeding [8, 10, 25-27]. In addition, cesarean section delivery, preterm delivery, infant health status and neonatal intensive care unit NICU admission were reported to impact mothers’ attitude to breastfeeding in Jordan [19, 25].

However, there is scarcity in research addressing factors that determine mothers’ attitude toward breastfeeding in Jordan. Hence, the purpose of this study was to assess mothers’ attitude toward breastfeeding, and to explore factors that may influence and predict this attitude among mothers.

Methods

Study Design and setting

This observational cross-sectional, hospital-based study was conducted to examine the attitudes of mothers in Jordan toward breastfeeding. Postnatal mothers were recruited from the maternity ward of a referral tertiary teaching hospital in Amman, the Capital city of Jordan, during the study period January 2020 to May 2021. This hospital is an accredited Baby-friendly hospital since June 2018 and has approximately 3000 births annually.

COVID lockdown and responsive adjustments at the study setting

Despite the hospital's high patient population, data collection was time-consuming because of the Covid-19 epidemic. During the pandemic, hospital admission and patient access were regularly limited when infected patients or personnel were reported.

Population and Sample

The target population for the current study was postnatal women in the maternity ward. The sample size was calculated using a Confidence Interval of 95% and a Margin of Error of 5%. A sample size of at least 290 was found to be adequate.

Study Instrument

The questionnaire for this study consisted of four parts. Iowa Infant Feeding Attitude Scale (IIFAS) [28], a validated tool used to measure attitudes of mothers toward infant feeding. It includes 17 items with a 5-point Likert scale ranging from 1 (strongly disagree) to 5 (strongly agree). Eight statements indicate positive attitude toward breastfeeding and nine statements indicate positive attitude toward formula feeding, the latter were reverse-scored before calculating the total scores of all items. An example for attitude is: "Formula-feeding is more convenient than breastfeeding", and "Breastfeeding increases mother-infant bonding". The total IIFAS score can range from 17 to 85 with higher scores reflecting more positive attitude towards breastfeeding. The total IIFAS is further classified as positive to breastfeeding (a score of 70 - 85), neutral (a score of 49 - 69) and positive to formula feeding (a score of 17-48). The researcher used a validated Arabic version of the IIFAS was used after permission from the author [29]. The tool was pilot tested on 30 participants to check for clarity, response time, any potential problems with the questionnaire in addition to reliability. Cronbach's alpha analysis revealed good reliability of the IIFAS, with coefficient equals to 0.72. No major changes were made based on the pilot test results.

Additionally, sociodemographic characteristics of mothers, and pregnancy and delivery information which includes mode of delivery, gestational age, the presence of complications during pregnancy or delivery, admission of the baby to neonatal intensive care unit (NICU), and receiving antenatal counseling for breastfeeding were all collected. The fourth aspect consisted of five questions to assess breastfeeding practices, which was created by the researcher abased on the Centers for Disease Control and Prevention (CDC) guidelines [28]. These include items about intention to breastfeed, willingness to breastfeed exclusively, receiving counselling for breastfeeding and time of initiation of breastfeeding.

Data collection procedure

Research team, were trained in interviewing procedures, collected data from postpartum mothers who entered the hospital's maternity ward after delivery. Data collection took place in the period between January 2020 and May 2021, using a structured interviewer-administered questionnaire. Despite the high volume of patients in this hospital, data collection required a long time, because of the Covid-19 pandemic restrictions.

The research team approached mothers after delivery, introduced themselves, and offered them the opportunity to participate in the research study. Those willing to participate were given further details and a verbal consent was obtained. Medical students were responsible for data collection, where they asked mothers questions and mothers responded. Medical students entered the answers electronically. The response rate was 95% with 301 mothers completed the questionnaires to collect sociodemographic information and the IIFAS questions. Data related to the pregnancy and delivery outcomes were collected from the medical record. Each mother's electronic medical record was searched for information pertaining to the pregnancy and birth outcomes. Every questionnaire was checked for completeness before medical students left the

ward. During data collection, appropriate infection prevention control measures and principles relevant to COVID-19 were also addressed.

Ethical Considerations

The ethical committee (IRB) in the University of Jordan Hospital approved this study (no. 2019-229). All study protocols complied with the guidelines established by the institution's committee on research ethics. Participation in the study was voluntary, and verbal consent was obtained from participating mothers. Mothers were assured that the service they receive would not be affected by their decision to participate in the study. Data obtained were kept confidential, and were used strictly for the purpose of this research. Confidentiality of the data was preserved, data were stored in password-protected files in the computers of the research team, and were used only for the purpose of this research.

Analysis

The collected data were examined for completeness and consistency, and then data was cleaned. Statistical analysis were performed using Statistical Package for Social Sciences (SPSS) version 21[29]. Descriptive statistics were used to summarize the sociodemographic personal characteristics, pregnancy and delivery variables, and infant breastfeeding-related variables. In addition, the mean and standard deviation for individual items of IIFAS, and total score for the attitude toward breastfeeding for each respondent were calculated. To determine the specific factors that influence breastfeeding attitude among participating mothers in the current study, independent t-test and one-way ANOVA statistical tests were performed. Additionally, multiple linear regression was used to detect predictors of attitude of mothers toward infant feeding. The results were expressed as adjusted odds ratio (AOR) with 95% confidence intervals (CI) and p-values. P-values <0.05 were considered statistically significant.

Results

Data collected from 301 mothers after delivery were included in the analysis. Table 1 demonstrates the sociodemographic characteristics of participants in addition to pregnancy and delivery information. More than 40%of the study population were 30 years of age or older and more than 70% had up to three children (including the recently delivered infant). Over 75% of participants had a university degree or higher. A large proportion of the sample were employed (43.2%). More than 60% of mothers had a monthly family income of 450 Jordan Dinar or less, and the vast majority (93%) had health insurance. The most common pregnancy complication among mothers was gestational diabetes (16%), and 26% had preterm deliveries constitutes 26% of infants. More than half of all deliveries were by a cesarean section (56.2%) and 18% of infants were admitted to NICU (Table 1).

Table 1. Sociodemographic characteristics, pregnancy and delivery variables of mothers toward breastfeeding (N = 301)

Variable	Categories	N	%
**Age**	<24	25	8
	24-30	105	35
	31-35	103	34
	>35	68	23
**Parity**	1	84	27.9
	2-3	131	43.5
	>= 4	86	28.6
**Level of Education**	Primary or lower	10	3.3
	Secondary level	61	20.3
	University degree or above	137	45.5
	Graduate degree	93	30.9
**Smoking (n=291)**	Non-smoker	238	82
	smoker	15	5
	Previous smoker	38	13
**Working status**	Employed	130	43.2
	Unemployed	171	56.8
**Monthly family income** (n=179)	0-300 JD	32	17.9
	301-450 JD	79	44.1
	451-650 JD	53	29.6
	>651 JD	15	8.4
**Health Insurance**	Yes	279	93
	No	21	7

Variable	Categories	N	%
**Pregnancy Complications**	No	205	68.1
	Gestational diabetes	44	16.4
	Hypertension	17	5.7
	Preeclampsia	11	3.7
	Other complications	24	8
**Delivery Complications**	No	271	90
	Yes	30	10
**Gestational Age (mean=41.4 week)**	Preterm	78	26
	Term	222	74
**Mode of Delivery**	Vaginal birth	132	43.9
	Cesarean Section Delivery	169	56.1
**Admission to NICU**	No admission to NICU	247	82.0
	Admission to NICU	54	18.0

Findings regarding breastfeeding-related variables are presented in Table 2. Almost all (95.7%) of mothers intended to breastfeed their infants, but only 35.2% of them were willing to breastfeed exclusively. Only 40% of mothers received any breastfeeding counselling before or after delivery. Around 43% of mothers initiated breastfeeding during the first day after firth, while 28.6% did not initiate breastfeeding before discharge (Table 2).

Table 2. Breastfeeding- related variables of mothers (N = 301)

Variable	Categories	N	%
**Intention to Breastfeed**	Yes	288	95.7
	No	10	3.3
	Undetermined	3	1.0
**Willingness to Breastfeed Exclusively**	Yes	106	35.2
	No	146	48.5
	Undetermined	49	16.3
**Breastfeeding counseling received**	Yes/ prenatal	60	20
	Yes/ postnatal	60	20
	No	181	60
**Time of Initiation of Breastfeeding**	Day 1	130	43.2
	Day 2	53	17.6
	Day 3	32	10.6
	Did not initiate	86	28.6

The attitude of participants to infant feeding, as measured using the IIFAS after delivery, are demonstrated in Table 3. The statements with the highest mean were “Breastfeeding increases mother-

infant bonding" and "Babies fed breast milk are healthier". The statements with the lowest means were "Mothers who occasionally drinks alcohol should not breastfeed" and "Women should not breastfeed in public places". Overall mean attitude toward infant feeding was 3.95 ± 1.04 (out of 5) and the overall total (summed) attitude score for participants was 65±7.15 (out of 85). This indicates a neutral postnatal attitude to breastfeeding, with only 24.3% of mothers demonstrating positive attitude to breastfeeding after childbirth (total attitude score ≥70 out of 85) (Table 3).

Table 3. Item scores of mothers' postnatal attitudes to breastfeeding (N=301)

Attitude statement	Likert Scale Responses* (%)					Item Mean ±SD
	1	2	3	4	5	
Benefit of breast milk last only until weaning **	48.8	4.98	11.63	7.3	27.2	3.41±1.74
Formula milk is more convenient than breastfeeding **	39.8	4.32	8.3	4.65	29.2	3.45±1.77
Breastfeeding increases mother-infant bonding	1.99	0.0	1.33	0.66	96.0	4.89±0.61
Breast milk is lacking in iron **	49.2	6.64	22.6	5.6	15.9	3.67±1.51
Formula-fed babies are more likely to be overfed	6.97	3.99	10.3	4.32	73.0	4.29±1.29
Formula-feeding is the better choice if a mother plans to work outside to work **	12.3	3.99	19.3	12.3	43.5	2.32±1.43
Mothers who formula feed miss one of the greatest joys of motherhood	5.6	0.99	3.65	7.3	79.0	4.51±1.12
Women should not breastfeed in public places **	10.9	4.98	12.3	4.3	63.0	2.01±1.48
Babies fed breast milk are healthier	4.3	1.33	3.65	5.65	84.0	4.63±0.99
Breastfed babies are more likely to be overfed **	76.7	6.64	8.64	2.66	5.3	4.47±1.10
Fathers feel left out if a mother breastfeeds **	45.5	7.97	19.6	8.64	14.2	3.69±1.49
Breast milk is the ideal food for babies	1.66	0.0	1.33	2.32	94.3	3.85±0.58
Breast milk is more easily digested than formula	1.99	1.33	4.32	2.66	89.0	4.74±0.81
Formula is as healthy for an infant as breast milk **	73.1	9.3	12.6	2.66	2.32	4.48±0.97
Breastfeeding is more convenient than formula feeding	26.2	6.31	11.3	4.98	51.1	3.48±1.73
Breast milk is less expensive than formula	6.3	1.66	3.0	2.2	86.7	4.62±1.73
Mothers who occasionally drinks alcohol should not breastfeed**	6.97	1.66	3.32	2.66	85.4	1.42±1.12

Overall mean attitude toward infant feeding = 3.95 ± 1.04

Total (summed) attitude score for participants was 65±7.15 (out of 85)

* 1= Strongly disagree, 2= Disagree, 3= Neutral, 4= Agree, 5= Strongly agree
**Statement with reverse score

Table 4 displays the relationship between mother's total attitude toward breastfeeding and personal characteristics, pregnancy and delivery variables, using t-test and ANOVA. Results show that attitude toward breastfeeding was significantly higher among women with higher income (p = 0.048), who had pregnancy complications (p = 0.049), with delivery complications (p = 0.008), who had preterm infants (p = 0.042), who intended to breastfeed (p = 0.002) and who were willing to breastfeed exclusively (p = 0.005).

Table 4. Relationship between sociodemographic characteristics, pregnancy and delivery outcomes and total attitudes score of mothers toward breastfeeding (N = 301)

Variable	Categories	N	%	Total Attitude Score [a]	*P*-value
**Age (mean=** 29.67, sd=)	<24	25	8	67.9	0.16[b]
	24-30	105	35	64.9	
	31-35	103	34	64.3	
	>35	68	23	65.0	
**Parity**	1	84	27.9	65.4	
	2-3	131	43.5	64.7	0.42 [B]
	≥4	86	28.6	66.5	
**Level of Education**	Primary or lower	10	3.3	60.6	
	Secondary level	61	20.3	64.0	0.12 [B]
	University degree or above	137	45.5	65.2	
	Graduate degree	93	30.9	65.7	
**Working status**	Employed	130	43.2	65.07	0.84[c]
	Unemployed	171	56.8	64.9	
**Monthly family income**	0-300 JD	32	17.9	62.5	
(n=179)	301-450 JD	79	44.1	64.6	**0.048** [B]
	451-650 JD	53	29.6	65.5	
	>651 JD	15	8.4	68.1	
**Pregnancy Complications**	No	205	68.1	64.5	**0.049** [c]
	Yes	96	31.9	66.1	
**Delivery Complications**	No	271	90	64.6	**0.008** [c]
	Yes	30	10	68.0	
**Gestational Age**	Preterm	78	26	66.4	**0.042** [c]
**(mean=41.4 week)**	Full term	222	74	64.5	
**Mode of Delivery**	Vaginal birth	132	43.9	64.5	0.41 [B]
	Planned CS	81	26.9	64.9	
	Emergency CS	88	29.2	65.8	

Variable	Categories	N	%	Total Attitude Score [a]	*P*-value
**Admission to NICU**	No admission to NICU	247	82.0	64.8	0.36 [B]
	Admission to NICU	54	18.0	65.6	
**Intention to Breastfeed**	Yes	288	95.7	65.2	**0.002** [B]
	No	10	3.3	57.2	
	Undetermined	3	1.0	65.3	
**Willingness to Breastfeed Exclusively**	Yes	106	35.2	66.7	**0.005** [B]
	No	146	48.5	63.8	
	Undetermined	49	16.3	64.9	
**Breastfeeding counseling during antenatal care**	Yes/ prenatal	60	20	64.9	0.99 [B]
	Yes/ postnatal	60	20	64.9	
	No	181	60	64.9	
**Time of Initiation of Breastfeeding**	Day 1	130	43.2	65.3	0.51 [B]
	Day 2	53	17.6	65.6	
	Day 3	32	10.6	64.4	
	Did not initiate	86	28.6	64.0	
Mean for the sum of attitude score= 65.0 ± 7.15 out of 85 (range 37-81)					

[a] Total attitude score is the sum of all items scores (range from 17 to 85)
[B] One-way ANOVA
[c] Independent t-test

After checking all assumptions, a multiple linear regression model was conducted to test for the effect of a combination of independent variables (age, education, income, intention to breastfeed, gestational age and NICU admission) on the attitude toward breastfeeding. Of the five independent variables, income level and intention to breastfeed showed a significant unique contribution to attitude toward breastfeeding total score (t = 2.43, p = 0.016; and t= 1.99, p= 0.048 respectively), whereas the remaining independent variables had no significant unique contributions. The multiple-linear regression model was statistically significant (F = 5.72, p = .050), with model's R =0.42, and R^2 =0.176, meaning that the combination of age, education, income, gestational age, intention to breastfeed and NICU admission was able to explain 17.64% of variance in the attitude toward infant feeding score of mothers who gave birth in the current study (Table 5).

Table 5. Results of multiple linear regression for the impact of age, education, income, intention to breastfeed, gestational age and NICU admission on mothers' positive attitude toward breastfeeding (N-301).

Variable	Unstandardized Coefficient (B)	Standardised Coefficient (β)	t	P-value	95% CI
**Age**	- 0.019	-0.014	- 0.18	0.860	- 0.23 – 0.19
**Education**	1.46	0.086	1.12	0.264	-0.80 - 3.36
**Income**	1.61	0.19	2.43	**0.016**	0.30 – 2.91
**Intention to breastfeed**	5.13	1.52	1.99	**0.048**	0.05 – 10.20
**Gestational Age**	- 1.77	- 0.111	-1.35	0.79	- 4.36 – 0.82
**Neonatal ICU admission**	- 1.45	- 0.087	-1.16	0.250	-3.93 – 1.03

Discussion

The rates of breastfeeding are on decline globally including Jordan. Mothers' attitude toward breastfeeding is a vital factor that influences breastfeeding practice. This study aimed to assess mothers' attitude toward breastfeeding in Jordan, and to identify the determinants and predictors of this attitude. Results of this study exhibit that a small proportion (24.3%) of mothers in Jordan have positive attitude toward breastfeeding. This is high compared to 9.2% having positive attitude to breastfeeding in Lebanon and Qatar [23], but considerably lower than 72% having positive attitude in Jordan in 2020 [24], and 55% in India [30].

The total attitude score in this study was 65.0 ± 7.15, which is very similar to total IIFAS score (63.5 ± 4.67) reported previously among Jordanian mothers [19]. This score is low compared to the very positive attitude with a total score of 81.39 ± 8.35 in Jeddah, Saudi Arabia [25], and to 69.76 ± 7.75 in Spain. On the other hand, lower total scores were reported in Poland (63.12 ±

7.34), in Saudi Arabia (59.6 ± 7.3) [31], and neutral attitude to breastfeeding (30 out of 50) in Fiji [8].

Findings of this study pointed out multiple sociodemographic characteristics and delivery characteristics that were associated with mother's attitudes to infant feeding. In accordance with other studies, socioeconomic status and particularly higher income, was associated with more positive attitude to breastfeeding in Saudi Arabia [25], Fiji [8] and Lebanon and Qatar [23]. In fact, higher family income continued to be a predictor of more positive attitude to breastfeeding, even after controlling for all other variables. This indicates the multifaceted nature of the attitude toward breastfeeding, involving a wide array of social norms, culture and health care environment factors.

Interestingly, mothers who had pregnancy and delivery complications had significantly more positive attitude to breastfeeding. Similarly, positive attitude to breastfeeding was associated with preterm delivery in this study. This contradicts earlier findings, where mothers of preterm infants were less likely to have positive attitude to breastfeeding [19], while other studies reported no association [23-25]. Our finding may reflect the psychological response of mothers in their effort to compensate their newborns with breastfeeding to support their growth with love and care. In addition, our results indicated that NICU admission did not negatively influence postpartum attitude to breastfeeding as expected, which implies that NICU of the baby was not a barrier to breastfeeding among mothers in this study. Although admission to NICU was associated with a significant delay in the initiation of breastfeeding to second or third day after childbirth, it did not prevent initiation. This may be because this hospital is baby-friendly, and the healthcare providers do encourage and support mothers to initiate breastfeeding after birth.

Despite the fact that no association was found in this study between working status or employment and attitude to breastfeeding, other studies reported a negative association, where employment was a barrier to breastfeeding [7, 13, 23-27, 32]. According to Al Tamimi et.al., 30% of the mothers in their study in Jordan, attributed premature cessation of breastfeeding to work [27]. In addition, Khasawneh et.al. indicated the short maternity leave as a prominent reason for non-exclusive feeding in Jordan [32]. The majority of mothers in the current study believed that mothers who were going back to their employment after childbirth, should choose formula feeding. Nevertheless, there was no difference in the mean attitude score between mothers who work and those who do not work, nor in their time of initiation of breastfeeding before discharge from hospital. This may suggest the more or less breastfeeding-friendly behavior that mothers perceive in many workplaces in Jordan.

We did not find any difference between mothers who received prenatal or postnatal counselling about breastfeeding and those who did not receive any counselling. In addition, no association was found between attitude and early initiation of breastfeeding. This is in line with findings of Khasawneh et.al., where counselling about breastfeeding by healthcare providers did not influence breastfeeding practice [24]. This may suggest that education and providing information about breastfeeding during pregnancy or just after delivery is not enough to influence mothers' attitude to breastfeeding.

This study demonstrated that mothers with intention to breastfeed and those who are willing to breastfeed exclusively had more positive attitude to breastfeeding. In fact, intention to breastfeed remained as a significant predictor even after controlling for other variables. This aligns with the findings from a number of studies in the literature [7, 23, 24], where associations were reported between planning and intending to breastfeed and attitude. Intention to breastfeed echoes mothers' attitude to breastfeeding, which depends on the deeply ingrained cultural and

social values that mothers have acquired through their life experiences. Therefore, inspiring mothers to breastfeed should be a component of the early stages of an antenatal care program or during planning for pregnancy.

To shape attitude to breastfeeding in mothers , it is essential to build more positive attitude to breastfeeding among husbands, mothers, mothers in law and friends of pregnant women [33]. Friends and family, among others in the community have a critical role in supporting, assisting, encouraging and motivating mothers to initiate and continue breastfeeding after childbirth, and their own attitudes to breastfeeding positively influence mothers' breastfeeding decisions and practices. They can effectively help mothers conquer barriers that may arise along the breastfeeding journey.

This study is the first to explore postnatal mothers' attitudes to breastfeeding in Jordan in addition to the influence of validated pregnancy and delivery outcomes on this attitude. Results of this study added insight to the evolving body of literature about mothers' breastfeeding attitudes in Jordan.

Limitations

Nevertheless, there are some limitations of this study. First, the main challenge was that data collection started just before Covid-19 pandemic, which interrupted and delayed the process of data collection. There was the lock down initially, followed by limitation to admissions to the wards during that period. Additionally, temporary closure of the ward when cases were identified limited access of the research team for data collection. Thus, the sample size is less than planned beforehand. Second, results may not be generalizable to mothers in Jordan, since our sample did not include mothers with no health insurance who gave birth in private hospitals. In addition, the study was carried out in a teaching hospital, which is certified as a baby-friendly hospital.

Therefore, results may have restricted generalizability to the population of mothers in Jordan who receive services in non-baby-friendly facilities. Third, the interviewer-administered way of data collection for the attitude, may have resulted in social desirability bias in responses, despite the training provided for the interviewers to reduce this bias.

Recommendations

Future research may explore women's attitudes before pregnancy, during pregnancy, and after delivery in order to investigate the effect of pregnancy and delivery experience on breastfeeding attitude. More qualitative studies can be of great benefit in exploring breastfeeding attitudes in the population in Jordan.

There is an immediate need for Interventions in Jordan to improve the rate of breastfeeding. Public policies can be implemented to increase the availability of designated breastfeeding places in workplaces, public places, parks, shopping malls, restaurants and health care facilities that are breastfeeding-friendly, prepared with a suitable place for breastfeeding. Policies and laws on marketing formula milk and breastmilk substitutes, paid maternity leave, availability of day care facilities, breastfeeding breaks for employed mothers, should all be enforced. Workplaces should encourage mothers to breastfeed their infants, and even reward them for adhering to this practice. Since the majority of women in Jordan give birth in private hospitals, more private hospitals better be encouraged to join the Baby Friendly Hospitals initiative. Interventions are specially needed to positively impact attitude to breastfeeding in low income communities, targeting women, their husbands, families and friends. Their support and encouragement is necessary for mothers to start and continue breastfeeding.

Health care students in all disciplines must be educated on breastfeeding. In addition, public education is essential to support and encourage woman's decision to breastfeed. To achieve that, initiatives must clearly communicate the benefits and importance of breastfeeding to the people [34], against the strong marketing for breastmilk substitutes by the companies. Well-designed interventions for improving breastfeeding counselling by health care providers after birth is vital. Maternity nurses can be very effective in educating, supporting and encouraging women to breastfeed. Special counselling provided to mothers who had cesarean delivery and who have newborns in the NICU is essential. Policies and laws on marketing formula milk and breastmilk substitutes, paid maternity leave, availability of day care facilities, breastfeeding breaks for employed mothers, should all be enforced. Workplaces should encourage mothers to breastfeed their infants, and even reward them for adhering to this practice.

Conclusion

To conclude, the determinants of mothers' attitude to breastfeeding was explored, in an effort to improve the rate of breastfeeding in Jordan. This study found that the mothers in Jordan have neutral attitude toward breastfeeding. In addition, it revealed the need for adopting various strategies for stimulating more positive attitude to breastfeeding on the population level, and especially people with low income, to optimize breastfeeding rates in Jordan. Healthcare professionals at all level of the health care system must play a vital role in promoting breastfeeding. Policy level interventions related to workplaces are extremely important in order to minimize the impact of employment as a barrier breastfeeding. Findings of this study can be instrumental for health care providers and policy makers in Jordan in their intentional efforts to promote breastfeeding on the population level.

Acknowledgements

we would like to acknowledge Salameh Halaseh and Murad Haddad for data entry and cleaning.

Also, Bdour Abdallat, Layan Ayesh, and Donia Jaber for their valuable efforts in data collection.

References:

[1] C. G. Victora *et al.*, "Breastfeeding in the 21st century: epidemiology, mechanisms, and lifelong effect," (in eng), *Lancet,* vol. 387, no. 10017, pp. 475-90, Jan 30 2016, doi: 10.1016/s0140-6736(15)01024-7.

[2] R. A. Raheem, C. W. Binns, and H. J. Chih, "Protective effects of breastfeeding against acute respiratory tract infections and diarrhoea: Findings of a cohort study," *Journal of paediatrics and child health,* vol. 53, no. 3, pp. 271-276, 2017.

[3] U. WHO, "Increasing commitment to breastfeeding through funding and improved policies and programmes: Global breastfeeding scorecard 2019," ed, 2019.

[4] U. N. C. s. F. (NUNICEF), "Breastfeeding: A Mother's Gift for, for Every Child," 2018. [Online]. Available: https://www.unicef.org/media/48046/file/UNICEF_Breastfeeding_A_Mothers_Gift_for_Every_Child.pdf

[5] W. H. Organization, "WHO. European Region has lowest global breastfeeding rates. 2015;[citado 2015 dez 18]," ed.

[6] X. Cai, T. Wardlaw, and D. W. Brown, "Global trends in exclusive breastfeeding," (in eng), *Int Breastfeed J,* vol. 7, no. 1, pp. 12-12, 2012, doi: 10.1186/1746-4358-7-12.

[7] Y. Alnasser *et al.*, "Impact of attitude and knowledge on intention to breastfeed: Can mHealth based education influence decision to breastfeed exclusively?," *Annals of Medicine and Surgery,* vol. 35, pp. 6-12, 2018/11/01/ 2018, doi: https://doi.org/10.1016/j.amsu.2018.09.007.

[8] S. P. Temoirokomalani M. D, Khan S. , "Knowledge, Attitude and Practices of Breastfeeding Among Mothers of Children Under 6 Months of Age in Suva, Fiji. ," *Curr Res Nutr Food Sci* vol. 9, no. 3, 2021, doi: http://dx.doi.org/10.12944/CRNFSJ.9.3.27

[9] M. I. Al Ketbi, S. Al Noman, A. Al Ali, E. Darwish, M. Al Fahim, and J. Rajah, "Knowledge, attitudes, and practices of breastfeeding among women visiting primary healthcare clinics on the island of Abu Dhabi, United Arab Emirates," *Int Breastfeed J,* vol. 13, no. 1, p. 26, 2018/07/03 2018, doi: 10.1186/s13006-018-0165-x.

[10] M. Alshebly and B. Sobaih, "Attitudes of Saudi mothers towards breastfeeding," *Sudanese journal of paediatrics,* vol. 16, no. 1, p. 31, 2016.

[11] J. Abuidhail, H. Al-Modallal, R. Yousif, and N. Almresi, "Exclusive breast feeding (EBF) in Jordan: Prevalence, duration, practices, and barriers," *Midwifery,* vol. 30, no. 3, pp. 331-337, 2014/03/01/ 2014, doi: https://doi.org/10.1016/j.midw.2013.01.005.
[12] N. H. Mohd Shukri, J. Wells, and M. Fewtrell, "Differences in maternal characteristics and their associations with breastfeeding attitudes among primiparous mothers," *Midwifery,* vol. 95, p. 102931, 2021/04/01/ 2021, doi: https://doi.org/10.1016/j.midw.2021.102931.
[13] M. Hasan, M. N. Hassan, M. S. I. Khan, M. A. Tareq, and M. S. Afroj, "Prevalence, knowledge, attitudes and factors associated with exclusive breastfeeding among mothers in Dhaka, Bangladesh: A cross-sectional study," *Population Medicine,* journal article vol. 3, no. September, pp. 1-7, 2021, doi: 10.18332/popmed/140132.
[14] O. G. Shobo, N. Umar, A. Gana, P. Longtoe, O. Idogho, and J. Anyanti, "Factors influencing the early initiation of breast feeding in public primary healthcare facilities in Northeast Nigeria: a mixed-method study," (in eng), *BMJ Open,* vol. 10, no. 4, p. e032835, Apr 20 2020, doi: 10.1136/bmjopen-2019-032835.
[15] F. Roostaee *et al.*, "Breast-feeding Continuation in South-Eastern of Iran: the Associated Factors," (in eng), *Med Arch,* vol. 69, no. 2, pp. 98-102, Apr 2015, doi: 10.5455/medarh.2015.69.98-102.
[16] S.-F. Yang, Y. Salamonson, E. Burns, and V. Schmied, "Breastfeeding knowledge and attitudes of health professional students: a systematic review," *Int Breastfeed J,* vol. 13, no. 1, p. 8, 2018/02/20 2018, doi: 10.1186/s13006-018-0153-1.
[17] V. Namasivayam *et al.*, "Association of prenatal counselling and immediate postnatal support with early initiation of breastfeeding in Uttar Pradesh, India," (in eng), *Int Breastfeed J,* vol. 16, no. 1, p. 26, Mar 16 2021, doi: 10.1186/s13006-021-00372-6.
[18] N. C. Bartle and K. Harvey, "Explaining infant feeding: The role of previous personal and vicarious experience on attitudes, subjective norms, self-efficacy, and breastfeeding outcomes," (in eng), *British journal of health psychology,* vol. 22, no. 4, pp. 763-785, 2017, doi: 10.1111/bjhp.12254.
[19] G. M. A. Shosha, "The influence of infants' characteristics on breastfeeding attitudes among Jordanian mothers," *Open Journal of Nursing,* vol. 5, no. 04, p. 295, 2015.
[20] M. Khassawneh, Y. Khader, Z. Amarin, and A. Alkafajei, "Knowledge, attitude and practice of breastfeeding in the north of Jordan: a cross-sectional study," *Int Breastfeed J,* vol. 1, no. 1, p. 17, 2006/09/23 2006, doi: 10.1186/1746-4358-1-17.
[21] L. Hamze, D. Carrick-Sen, Z. Zhang, Y. Liu, and J. Mao, "Maternal attitude towards breastfeeding: A concept analysis," *British Journal of Midwifery,* vol. 26, no. 7, pp. 462-469, 2018, doi: 10.12968/bjom.2018.26.7.462.
[22] L. K. Twells *et al.*, "Assessing Infant Feeding Attitudes of Expectant Women in a Provincial Population in Canada: Validation of the Iowa Infant Feeding Attitude Scale," (in eng), *J Hum Lact,* vol. 32, no. 3, pp. Np9-np18, Aug 2016, doi: 10.1177/0890334414559647.
[23] F. Naja *et al.*, "Prenatal breastfeeding knowledge, attitude and intention, and their associations with feeding practices during the first six months of life: a cohort study in Lebanon and Qatar," (in eng), *Int Breastfeed J,* vol. 17, no. 1, pp. 15-15, 2022, doi: 10.1186/s13006-022-00456-x.
[24] W. Khasawneh, K. Kheirallah, M. Mazin, and S. Abdulnabi, "Knowledge, attitude, motivation and planning of breastfeeding: a cross-sectional study among Jordanian women," *Int Breastfeed J,* vol. 15, no. 1, p. 60, Jul 1 2020, doi: 10.1186/s13006-020-00303-x.
[25] B. A. Mohammed and S. A. Soliman, "Mothers' Attitudes toward Breastfeeding and Their Association with Infants' Characteristics," *J Nurs Heal Sci,* vol. 7, no. 3, pp. 60-69, 2018.
[26] R. Yasser Abulreesh *et al.*, "Attitudes and Barriers to Breastfeeding among Mothers in Princess Nourah Bint Abdulrahman University, Riyadh, Kingdom of Saudi Arabia," *The Scientific World Journal,* vol. 2021, p. 5585849, 2021/08/02 2021, doi: 10.1155/2021/5585849.

[27] E. Altamimi, R. Al Nsour, D. Al dalaen, and N. Almajali, "Knowledge, Attitude, and Practice of Breastfeeding Among Working Mothers in South Jordan," *Workplace Health & Safety,* vol. 65, no. 5, pp. 210-218, 2017, doi: 10.1177/2165079916665395.

[28] "Questionnaires: Breastfeeding and Infant Feeding Practices." https://www.cdc.gov/breastfeeding/data/ifps/questionnaires.htm (accessed August 30, 2021.

[29] *IBM SPSS Statistics for Windows*. ((2011)). [Online]. Available: Retrieved from https://hadoop.apache.org

[30] M. Krishnendu and G. Devaki, "Knowledge, attitude and practice towards breasfeeding among lactating mothers in rural areas of Thrissur District of Kerala, India: a cross-sectional study," *Biomedical and Pharmacology Journal,* vol. 10, no. 2, pp. 683-690, 2017.

[31] R. Yasser Abulreesh *et al.*, "Attitudes and Barriers to Breastfeeding among Mothers in Princess Nourah Bint Abdulrahman University, Riyadh, Kingdom of Saudi Arabia," (in eng), *ScientificWorldJournal,* vol. 2021, p. 5585849, 2021, doi: 10.1155/2021/5585849.

[32] W. Khasawneh and A. A. Khasawneh, "Predictors and barriers to breastfeeding in north of Jordan: could we do better?," (in eng), *Int Breastfeed J,* vol. 12, p. 49, 2017, doi: 10.1186/s13006-017-0140-y.

[33] J. T. Wallenborn, D. C. Wheeler, J. Lu, R. A. Perera, and S. W. Masho, "Importance of Familial Opinions on Breastfeeding Practices: Differences Between Father, Mother, and Mother-in-Law," (in eng), *Breastfeed Med,* vol. 14, no. 8, pp. 560-567, Oct 2019, doi: 10.1089/bfm.2019.0049.

[34] U. a. WHO, "Breastfeeding Advocacy Initiative: for the Best Start in Life.

," 2015. [Online]. Available: https://apps.who.int/iris/bitstream/handle/10665/152891/WHO_NMH_NHD_15.1_eng.pdf;sequence=1
