# Supplementary material for: Determinants of breastfeeding attitudes of mothers in Jordan: A cross-sectional study
Source: PLoS One. 2023 May 5;18(5):e0285436. doi: 10.1371/journal.pone.0285436 (PMC10162564; doi:10.1371/journal.pone.0285436)
Supplement: Attachment — Submitted filename: Response to Reviewers.docx [file pone.0285436.s002.docx]

**PONE-D-22-35675**

**Determinants of Breastfeeding Attitudes of Mothers in Jordan: A Cross-sectional Study**

**PLOS ONE**

**Author responses are marked in BLUE**

**Additional Editor Comments (if provided):**

1) ABSTRACT:
¬ No need for details of methodology in the abstract. Only main points. It should not include the type of statistical analysis. Methodology has to mention the elements included as measuring parameter but without details.

Names of statistical tests have been removed.
¬
2) INTRODUCTION:
¬ Newest Global/ Regional/ Jordan prevalence of mothers’ attitude toward breastfeeding should be stated.

I have added and included what indicates the state of attitude to breastfeeding globally, regoionally, and in Jordan.

¬ The current situation of other developed and developing countries should also be added.

The previous point included developed and developing countries.

¬ The benefits of conducting the study to the community should be explained.

Statements have been added to indicate benefits to the community, such as: “Advancing better understanding of the country-specific determinants and predictors of women’s attitudes toward breastfeeding is critical for development of effective health care programs, and for informing health care providers and policy makers in Jordan.”

3) METHODS:

¬ The characteristics of the study participants should be mentioned as inclusion criteria and exclusion criteria (if any).

There were no inclusion or exclusion criteria

¬ How long did it take to complete each questionnaire?

The answer was added: it took 10 minutes to answer each questionnaire.

¬ What types of data (quantitative and/or qualitative) were used?

Answer provided: all data were quantitative .
¬ Definitions as postnatal period, Infant, exclusive breastfeeding, ……. etc. should be mentioned.

Definitions were provided in the appropriate place in the methods.
¬ How did the authors get the study subjects? They have to clearly address their sampling technique?

Details were provided.
¬ Authors should include a reference for using the stated formula in calculating the sample size. Furthermore, the basis of sample size calculation should be mentioned to know the confidence level and the margin of error. Please give a justification for the sample size. How did you determine the sample size?

A reference was provided for the sample size calculator used.
4) RESULTS:

¬ In table 3, the sentence: “Overall mean attitude toward infant feeding was 3.95 ± 1.04 (out of 5)” need to be explained.

(out of 5) was deleted. No need for it since it is made clear in the table that 5-point Likert was used.

5) DISCUSSION:
¬ Discuss by using the scientific reasoning the mothers’ attitude toward breastfeeding and the determinants of this attitude in other developing and developed countries with similar context. The manuscript could be greatly strengthened if the authors could compare the findings of the study with other findings and state the reasons for the strengths and weaknesses in each section.

Comparisons with developed and developing countries has bees introduced.

6) CONCLUSION:
¬ It should be specific and based on the findings of the study.
I have deletes sentences not based on results of the study.

[

**Reviewer #1: Response to reviewers**
The authors have submitted a manuscript titled “Determinants of breastfeeding attitudes of mothers in Jordan” This study is very useful and adds a significant value in the maternal and child health system of Ethiopia regarding the perception and attitudes of breastfeeding. However, I will recommend some of the minor suggestions to make this manuscript better.

1)In addition, exclusive breast feeding for six months, continued for two years and beyond with the provision of safe and appropriate complementary foods are the most important strategies for promoting child survival and health [4]. However, the benefits of breastfeeding are underestimated, and the proportion of mothers who breastfeed their new-borns is way below the recommended levels globally and Jordan is not an exception [5, 6].
In this statement Although importance of breastfeeding has been explained quite evidently, addition of relevant information in context of exclusive breastfeeding globally as well as regionally should be explained

Breastfeeding tares in Jordan and in the region have been added.

2) These factors include mothers’ age, income, education, mode of delivery and availability of breastfeeding counselling before, during and after delivery, and past breastfeeding experience [7-13].
How the mentioned socio-demographic factors are crucial for breastfeeding experience should be elaborated. For ex- how education is important for prevalence of exclusive breastfeeding.

More explanation has been added about the positive or negative effect of certain factors on breastfeeding attitudes.

3) In addition, health professionals’ attitudes, type of health care facility where delivery occurred, and admission of the infant in neonatal intensive care unit (NICU) influence the process of breastfeeding by women [9, 14-17
Justification of how healthcare professionals’ attitude or type of care received is important for perception of breastfeeding

Explanation was provided.

4) The target population for the current study was postnatal women in the maternity ward. The sample size calculated using a confidence interval of 95% and a margin of error of 5%. A sample size of atleast 290 was found to be adequate
How did the authors have selected the samples and how they were found to be adequate have they taken any reference from similar studies of same population or whether they have done any sample size calculation.

I have added explanation and reference for the sample size calculation, and provided rationale for the sample selection and size.

5) Friendly, prepared with a suitable place for breastfeeding. Policies and laws on marketing formula milk and breastmilk substitutes, paid maternity leave, availability of day care facilities, breastfeeding breaks for employed mothers, should all be enforced.

In above statement Information regarding milk bank for those mothers who are unable to breastfeed the child due to less milk production.

The idea of a “milk bank” may not be appropriate to propose in a country like Jordan, due to cultural and religious issues.

6) Special counselling provided to mothers who had caesarean delivery and who have new-borns in the NICU is essential. Policies and laws on marketing formula milk and breastmilk substitutes, paid maternity leave, availability of day care facilities.
Importance of propagation of importance of breastfeeding through mhealth.,

I have added the concept of importance of breastfeeding through maternal health services.

**Reviewer #2:**

This is interesting research. The authors need a little help in improving the writing and need to get a statistician on-board to help with the statistical test and appropriate interpretation. After making some improvements, the manuscript can get to a point where it is publishable in this journal.

I would like to thank Reviewer #2 for the very constructive comments and suggestions for improving the manuscript.

Abstract
The first two statements in the abstract are not objective. They are also not presented in a scientific manner, but rather as a more casual and social statement.

First two sentences replaced by a more objective sentence.

For the analysis, the authors use a multiple linear regression model to assess the association between attitude scores from the IIFAS questionnaire, and various maternal sociodemographic and pregnancy related characteristics. However, they report odds ratios, which is not the metric that results from a linear regression model. The authors need to get a statistician on their team.
That was an evident mistake. Any way, a statistician has been consulted and I have replaced multiple linear regression with logistic regression.

Introduction:
End of paragraph 2:
Non-scientific expression, and no references – “The proportion of mothers who breastfeed their newborns ‘is way below’ the recommended levels globally, and Jordan ‘is not an’ exception”. (correct usage: Jordan is no exception).

Valid references have been provided to indicate the low rates of breastfeeding globally.

The expression has been corrected to become: Jordan is no exception.

End of paragraph 3:
Incomplete sentence – “Furthermore, several studies have demonstrated that mothers’ attitudes toward breastfeeding is a significant predictor of their intention, initiation, and continuation of.”

True, sentence was incomplete. The missing word is added: breastfeeding.

Paragraph 4 and 5 needs to be tighter and better worded for a scientific journal. For example, ‘What determines women’s attitude towards breastfeeding?’ can be stated as ‘We sought to assess factors that determine women’s attitude towards breastfeeding’. Also, the authors do not build interest on the need for their research in an effective manner.
The question was deleted, and suggested sentence added. In addition to other changes to the paragraph.

Methods:
Study Design and setting
COVID lockdown and responsive adjustments at the study setting looks like an unnecessary sub-heading. These details can just be incorporated into the paragraph on setting.

I agree, the subheading was deleted and details included in setting.

Population and Sample
Sample size calculation: The Section on Population and Sample is too concise and needs further detail with respect to power and sample size calculations. Insufficient details are given, and the sentence ‘A sample size of at least 290 was found to be adequate’ is lacking. The authors need to explain what a sample size of 290 is adequate for. This section is a good place to discuss sample size and power calculations. What is the minimal difference that can be detected with a sample size of 290?

A new calculation for sample size was provided with reference, which is more detailed.

Study Instrument
This section is not presented with proper English grammar, punctuation, and sentence structure. It needs to be presented better. Some sentences in this section appear like bullet points instead of complete sentences. For example, “Iowa Infant Feeding Attitude Scale, a validated tool used to measure attitudes of mothers toward infant feeding.”.

The sentence has been completed; see track changes.

Other sentences are too long and need to be broken down. For example, “Eight statements indicate positive attitude toward breastfeeding and nine statements indicate positive attitude towards formula feeding, the latter were reverse-scored before calculating the total scores of all items”.

This very long sentence has been broken down into two sentences, and adjusted to be clearer.

Other statements that are grammatically incorrect include “The researcher used a validated Arabic version of the IIFAS was used after permission from the author”.

The sentence was corrected.

The second paragraph in this section includes a sentence that starts with “The fourth aspect”. This is confusing, and the authors did not explicitly indicate what the first three aspects were.

This has been correctd.

Also, the same sentence continues on to read ‘abased’ instead of ‘based’.

Abased is incorrect, it was replaced with “based”.

Data collection procedure
The first sentence has incorrect punctuation, lacks proper structure, and grammar.

Sentence was rearranged and edited.

Correct grammar would imply using ‘completing’ instead of ‘completed’ in the statement: “The response rate was 95%, with 301 mothers ‘completing’… questions”.

**This was corrected to “completing”**

Analysis
This section mentions, “To determine the specific factors that influence breastfeeding attitude among participating mothers in the current study, independent t-test and one-way ANOVA statistical tests were performed”. However, the results as presented in Table 4 can be more appropriately stated as follows: “Independent t-test and one-way ANOVA tests were performed to assess if the total attitude score towards breastfeeding different significantly across each of the measured patient characteristics”.

I replaces the old sentence with the one reviewer #2 suggested.

The section mentions the use of ‘multiple linear regression models’ but talks about expressing results as adjusted odds ratios with 95% CI. Logistic regression models produce odds ratios, and the statement used in this section is not congruent with the results produced in the subsequent section.

The comment was perfectly in place. I preferred to replace multiple linear regression with logistic regression, which was easier to interpret. Still. Results did not change, the same two variables stayed significant.

Results
It is more appropriate to use the term ‘presents’ than ‘demonstrates’ when talking about the tables. For example, ‘Table 1 presents sociodemographic characteristics…. information”.

Demonstrates was replaced with presents.

The sentence is not correctly constructed: “The most common pregnancy complication among mothers was gestational diabetes (16%), and 26% had preterm deliveries constitutes 26% of infants”.

The sentence was corrected: The most common pregnancy complication among mothers was gestational diabetes (16%), and 26% of mothers had preterm deliveries

The title for table 1 does not make sense.

Title has been corrected: **Sociodemographic characteristics, pregnancy and delivery variables of mothers participating in this study about attitudes toward breastfeeding (N = 301)**

The authors need to be consistent in the tables and in the text with regards to decimal places on percentages (e.g., use one decimal place throughout the manuscript for %s) and make sure the percentages reported in the table and the manuscript text match exactly.

The title for table 2 needs to be improved.

Title has been improved.: **Breastfeeding- related variables of mothers participating in this study about attitudes toward breastfeeding (N = 301)**

The sentence right above Table 2 reads ‘firth’ instead of ‘birth’.

The word was corrected: birth.

The IIFAS statement with the second highest mean score as mentioned in the text in the paragraph above table 3 does not match with what is presented in Table 3.

The corrected item was placed.

The statement “This indicates a neutral postnatal attitude to breastfeeding” is unsubstantiated based on a mean total summed attitude score of 65 and a standard deviation of 7.15. There needs to be a statistical test that proves this statement.

In study instrument section of the Methods: total attitude score was classified as positive to breastfeeding (a score of 70 - 85), neutral (a score of 49 - 69) and positive to formula feeding (a score of 17-48). This classification is added as a foot note beneath the table.

In the paragraph that starts with the statement ‘Table 4 displays’, the second sentence mentions ‘attitude towards breastfeeding was significantly higher’, but a higher attitude is not a valid statement. Perhaps a ‘higher score’ or a ‘more positive attitude’ would be a more suitable word.

This was corrected: higher total attitude score.

In the next paragraph right after Table 4, the authors list 6 independent variables, but the sentence reads, “Of the five independent variables”.

The paragraph was deleted, though the comment is valid.

The results as presented in Table 5 need to be presented better and the title for Table 5 needs to be more concise. It appears that the authors used a multiple linear regression model, with the participant’s ‘Total attitude score’ as the dependent variable, and age, education, income, intention to breastfeed, gestational age, and neonatal ICU admission as the independent variables. However, at least four of the independent variables would be categorical variables. The author needs to state which category was used as the reference, particularly for categories with more than two response options, example, Education. Furthermore, the authors do not interpret these coefficients in the results section, either by their unstandardized estimates, or standardized estimates. It must be pointed out that manuscripts don’t typically report standardized coefficients from a multiple linear regression model since they are not directly interpretable. Unstandardized coefficients use the natural unit of measure for the specific variable and can be easily interpreted, and are therefore, the only reported coefficients in scientific manuscripts. For example, based on the results in Table 5, a unit increase in income results in a 1.61-point increase in the attitude score. However, this interpretation would only apply if income was measured as a continuous variable. However, the authors did not state if income is a continuous variable in their model, and in case it was categorical, the reference category is not mentioned. This makes interpreting the coefficients difficult. Assuming that the model is specified correctly, the p-values would suggest the variables/maternal factors that are significantly associated with the total scores on the IIFAS questionnaire.

It seems that I did faced some problems interpreting results of multiple linear regression.

I have consulted statistician. I decided to replace multiple linear regression with logistic regression.

Finally, it appears that the authors are interpreting the standardized coefficients as odds ratios based on their statement in the abstract. This is incorrect, and a statistician would be a good addition to the team in improving this paper.

That was a mistake actually.

**Reviewer #3:**

Determinants of Breastfeeding Attitudes of Mothers in Jordan: A Cross-sectional Study
Revision of the study “Determinants of Breastfeeding Attitudes of Mothers in Jordan: A Cross-sectional Study”.
This study aims to investigate the Breastfeeding Attitudes of Mothers in Jordan; during the study period January 2020 to May 2021.
Evidence suggests that the breastfeeding attitudes are higher in low-income mothers and general people.
Limitations of the study are noted and discussed.
I think the paper covers an import area of research.
I have listed some specific comments below that the authors should take into account before this
work could be published.

Abstract:
“The mean attitude score for participants was 65.0 ±7.15, indicating a neutral attitude. Factors associated with positive breastfeeding attitude were high income (p=0.048), pregnancy complications (p= 0.049), delivery complications (p= 0.008), prematurity (p= 0.042), intention to breastfeed (p= 0.002) and willingness to breastfeed (p= 0.005). Multiple linear regression results indicated higher income and intention to breastfeed as predictors of positive breastfeeding attitude (AOR 0.019; 95% CI 0.30-2.91 and AOR 1.52; 95%CI 0.05-10.20 respectively).”
I believe focusing on these risk factors should be the main aim of the study. What do you
mean when you say: “positive breastfeeding attitude”?

“positive breastfeeding attitude” was corrected to become: Attitude is positive to breastfeeding”

Please specify if these were protective or risk factors.

Multiple linear regression was replaced with Logistic regression.

New analysis:

With binary logistic regression modelling, predictors of positive attitude to breastfeeding were highest income and willingness to breastfeed exclusively (AOR= 18.97, 95%CI= 3.00-124.11 and AOR= 3.49, 95%CI= 0.36-3.31 respectively). Looking at the odds ratios, attitude that is positive to breastfeeding was significantly higher among mothers with higher income and mothers who were willing to breastfeed exclusively.

Introduction:
1. Could you please add 2 or 3 references about the global scenario regarding Breastfeeding Attitudes of Mothers?

References have been added.

Method:
1. Which sampling technique authors were used?

I have added the reference for the sample size calculator used. I have elaborated more on the way women were included in the study.

Result:
The tables and figures are well described.

Titles of table 1 and table 2 have been revised.

Discussion:
Overall discussion was well written. Just add 2 or 3 references which is related to your significant results.

References have been added as recommended.
