# Supplementary material for: Determinants of breastfeeding attitudes of mothers in Jordan: A cross-sectional study
Source: PLoS One. 2023 May 5;18(5):e0285436. doi: 10.1371/journal.pone.0285436 (PMC10162564; doi:10.1371/journal.pone.0285436)
Supplement: Attachment — Submitted filename: Rebuttal letter (revision 2).docx [file pone.0285436.s004.docx]

**PONE-D-22-35675R1**

**Determinants of Breastfeeding Attitudes of Mothers in Jordan: A Cross-sectional Study**

**PLOS ONE**

Journal Requirements:

Please review your reference list to ensure that it is complete and correct. If you have cited papers that have been retracted, please include the rationale for doing so in the manuscript text, or remove these references and replace them with relevant current references. Any changes to the reference list should be mentioned in the rebuttal letter that accompanies your revised manuscript. If you need to cite a retracted article, indicate the article’s retracted status in the References list and also include a citation and full reference for the retraction notice.

**References have been revised and corrected.**

Additional Editor Comments (if provided):

Reviewer #2 comment which was not corrected:
The authors need to be consistent in the tables and in the text with regards to decimal places on percentages (e.g., use one decimal place throughout the manuscript for %s) and make sure the percentages reported in the table and the manuscript text match exactly.

**I have made all numbers and percentages with one decimal point. Except p-values and confidence intervals, which I thought that two decimal points would be more appropriate. I have also referred to papers published in PLOS ONE and found p-vales and CI presented with two decimal points.**

Make sure you thoroughly address the reviewers' comments and concerns and ensure the manuscript is free of any editorial or grammatical errors.

[Note: HTML markup is below. Please do not edit.]

Reviewers' comments:

Reviewer's Responses to Questions

**Comments to the Author**

1. If the authors have adequately addressed your comments raised in a previous round of review and you feel that this manuscript is now acceptable for publication, you may indicate that here to bypass the “Comments to the Author” section, enter your conflict of interest statement in the “Confidential to Editor” section, and submit your "Accept" recommendation.

Reviewer #4: All comments have been addressed

Reviewer #5: (No Response)

Reviewer #6: (No Response)

Reviewer #7: All comments have been addressed

2. Is the manuscript technically sound, and do the data support the conclusions?

The manuscript must describe a technically sound piece of scientific research with data that supports the conclusions. Experiments must have been conducted rigorously, with appropriate controls, replication, and sample sizes. The conclusions must be drawn appropriately based on the data presented.

Reviewer #4: Yes

Reviewer #5: Yes

Reviewer #6: Partly

Reviewer #7: Yes

3. Has the statistical analysis been performed appropriately and rigorously?

Reviewer #4: Yes

Reviewer #5: Yes

Reviewer #6: I Don't Know

Reviewer #7: Yes

4. Have the authors made all data underlying the findings in their manuscript fully available?

The [PLOS Data policy](https://eur01.safelinks.protection.outlook.com/?url=http%3A%2F%2Fwww.plosone.org%2Fstatic%2Fpolicies.action%23sharing&data=05%7C01%7Cs.alkhaldi%40ju.edu.jo%7Ce86774f1c2ac48f3aa9208db236ed606%7C05405dba373c4e20a30e3e6fcf507cfe%7C0%7C0%7C638142731504372794%7CUnknown%7CTWFpbGZsb3d8eyJWIjoiMC4wLjAwMDAiLCJQIjoiV2luMzIiLCJBTiI6Ik1haWwiLCJXVCI6Mn0%3D%7C3000%7C%7C%7C&sdata=12RXCsO4x3JpMYGBZuUxSNj6ftX0tGSoFx%2BqnCDAe%2BQ%3D&reserved=0) requires authors to make all data underlying the findings described in their manuscript fully available without restriction, with rare exception (please refer to the Data Availability Statement in the manuscript PDF file). The data should be provided as part of the manuscript or its supporting information, or deposited to a public repository. For example, in addition to summary statistics, the data points behind means, medians and variance measures should be available. If there are restrictions on publicly sharing data—e.g. participant privacy or use of data from a third party—those must be specified.

Reviewer #4: No

Reviewer #5: Yes

Reviewer #6: (No Response)

Reviewer #7: Yes

**I have made the data set available with the last revision.**

5. Is the manuscript presented in an intelligible fashion and written in standard English?

PLOS ONE does not copyedit accepted manuscripts, so the language in submitted articles must be clear, correct, and unambiguous. Any typographical or grammatical errors should be corrected at revision, so please note any specific errors here.

Reviewer #4: Yes

Reviewer #5: No

Reviewer #6: No

Reviewer #7: Yes

6. Review Comments to the Author

**Reviewer #4:** The study involves analyzing data of breastfeeding attitudes of mothers in Jordan that gathered from one teaching hospital in Amman to examine the attitudes of mothers toward breastfeeding. The methods used a questionnaire according to Iowa Infant Feeding Attitude Scale to measure attitudes of mothers toward infant feeding. The results showed that the mean attitude score for participants was 65.0 ±7.15, indicating a neutral attitude. Factors associated with positive breastfeeding attitude were high income (p=0.048), pregnancy complications (p= 0.049), delivery complications (p= 0.008), prematurity (p= 0.042), intention to breastfeed (p= 0.002) and willingness to breastfeed (p= 0.005).

Comments and inquiries below are mostly minor: Please see the attached document:
1. Could you include how the participants were recruited? Did they receive hard copy, or they answered orally? Explain the method.

**Participants were recruited when they were attending the maternity ward after delivery (as explained in data collection section).**

**“Medical students were responsible for data collection, where they asked mothers questions and mothers responded.** **In compliance with infection control procedures, and for avoiding paper-based questionnaires, medical students entered the answers electronically immediately, using their mobile phones or tablets”.**

2. Was there a complete lockdown in the delivery section? If yes, mention the period.

**Lockdown occurred on and off according to covid-19 cases detected among patients or health care providers.**

3. Could you state the inclusion or exclusion criteria for selecting the participants?

**Women who were still under the effect of anesthesia, or who seemed very tired at the time of data collection were excluded.**

4. Was there an informed consent or not?

**Verbal consent was obtained from women as mentioned in the ethical considerations section.**

5. Did you use any sampling method? Like random or systemic sampling technique for selecting the participants.

**Convenience sample was used. All women available at the time of data collection were invited to participate.**

6. It looks like interview. Is that right? If yes, did all the patients answered verbally? How long was it take? If mixed, written or interview, what was the percentage of each. Did you record the interview?

**It was a structures interviewer-administered questionnaire.**

**Medical students were responsible for data collection, where they asked mothers questions and mothers responded. In compliance with infection control procedures, and for avoiding paper-based questionnaires, medical students entered the answers electronically immediately, using their mobile phones or tablets.**

**Interview was not recorded.**

7. In the data collection procedure section: line 9: Medical students entered the answers electronically. Do you mean for analyzing the data? Clarify.

T**hey asked the women questions, and entered the answers into the electronic questionnaire.**

8. Did you take the permissions from the patients before collecting these data? Explain

**“The research team attended the maternity ward whenever they were allowed to do so. They approached all mothers available in the ward at the time of their visit. They introduced themselves, and offered women the opportunity to participate in the research study. Women who were willing to participate were given further details and a verbal consent was obtained. “**

9. In the analysis section: In addition, the mean and standard deviation for individual items of IIFAS, and total score for the attitude toward breastfeeding for each respondent were calculated. How? Which test did you use?

**There was not test for calculating the mean.**

**In table 3, the mean for individual item was presented.**

**In table 4, the total attitude score for each respondent was calculated by adding up the scores for the 17 items for each respondent.**

10. In table 4: undetermined: Why, what was the reason? explain that in the footnote under the table.

**A footnote was added beneath the table indicating what undetermined means.**

11. Why are mothers more likely to use formula than breastfeed?

**I have added: “ This may be due to the belief that formula milk is an advanced combination of nutrients and that it is as good as breastmilk.”**

12. In the end of page 14: healthcare provider: Did they use ads for breastfeeding? What kind of encourage did they provide?

**They remind women and bring their attention to the importance of breastfeeding, and encourage them to initiate breastfeeding and continue.**

Reviewer #5: Comment” The characteristics of the study participants should be mentioned as inclusion criteria and exclusion. The authors responded “There were no inclusion or exclusion criteria” . It cannot be, there must be inclusion and exclusion criteria for every

**“A convenience sample was selected from the target population for the current study, which was all postnatal women attending the maternity ward after delivery, when the research team was available in the hospital, during the study period. Women who were still under the effect of anesthesia, or who seemed very tired at the time of data collection were excluded. study and yours of no exception “.**

Other comments by reviewers were endorsed adequately by the authors.
Further Comments:

Under Introduction: the statement “Yet, About 44% of infants 0–6 months old are exclusively breastfed over the period of 2015-2020 [4]. Where? I cannot access the link for reference 4.

**I did copy and paste the link and it opened with me. In the Global Health Observatory, chose the early initiation of breastfeeding indicator and it displays the statistics.**

Under Introduction: the statement “The decision about infant feeding seems to be made before childbirth” the authors quoted reference [21], however this conclusion was not mentioned in that article. Please revise.

**Naja et al. in their paper studied women’s “intention to breastfeed” using six questions. Their results indicated:**

**“For the intention to breastfeed, 43.4% of women had very strong intentions to breastfeed, 25.4% had strong intentions, 18.4% fair intentions and 12.7% weak intentions (Fig. 4d).”**

**“Both positive attitude towards breastfeeding and strong intention to breastfeed were associated with EBF at four months, breastfeeding at four months, and breastfeeding at six months (Table 6).”**

The objective of the study should be stated clearly at the end of the introduction.
**This i“Hence, in this study, we sought to assess factors that determine women’s attitude towards breastfeeding in Jordan.**”

What was the validity and reliability of IIFAS questionnaire?

**The IIFAS was prevalidated, and pilot test was performed and reliability was calculated (reference 28).**

Item 17 of IIFAS questionnaire is about drinking alcohol for breastfeeding women. I find it difficult to ask Muslim Jordanian women this question. Please address my concern.

**We did actually hesitate to keep this item about drinking alcohol, but then preferred to keep it. Since it can provide an insight into women’s perception about the influence of what they eat or drink on their infants.**

The authors stated that “ The researcher used a validated Arabic version of the IIFAS was used after permission from the author” and they quoted reference 29 for this statement. Indeed, the authors under reference 29 used a semi structured questionnaire survey and they did not use IIFAS questionnaire, please revise.

**You are totally right. I got permission from Charafedding from Lebanon, who did the validation for the Arabic version.**

Under data collection procedure: the authors sated that “response rate was 95% with 301 mothers completing the questionnaires” What was the dominator for 301 mothers?

**The total number of women approached was 316, out of which, 15 did not want to participate. Leading to a 95% response rate.**

The authors stated that “monthly family income of 450 Jordan Dinar or less” please convert this to US$ as readers are unfamiliar with Jordan Dinar.

**I added a foot note beneath table 1 (1 JD equals US$ 1.4)**

**Reviewer #6:** General comment:

Based on other studies on the determinants of breastfeeding, some of which are included in the manuscript bibliography, my question to the authors is: why was “knowledge of breastfeeding” not included in the study question? The Iowa Infant Feeding Attitude Survey (IIFAS) questionnaire used for data collection has items that can be better classified as “knowledge” rather than as “attitude”. The IIFAS tool developers could be asked as to whether they intentionally classified all survey items as assessment of “attitude” when in fact they appear to be testing “knowledge”.

**While some questions may seem like knowledge oriented, I think they do measure perceptions and attitudes more accurately.**

Parturient mothers may be less intimidated to answer questions on their “attitude” and may be more apt to accept participation in the study, as opposed to thinking that they would be tested and evaluated with a survey of their correct or incorrect “knowledge,” whereas attitudes are neither right or wrong. However, the research needs to determine if the items are truly measuring “attitude” or “knowledge.” The manuscript would be clearer to readers if the authors could discuss the issue of “knowledge” versus “attitude” in relation to intent and practice of breastfeeding, and why they have excluded the assessment of knowledge. There may be studies available in the literature that address that issue.

**This study did not aim to assess knowledge from the beginning. Our objective was to** **assess attitude to breastfeeding, and the IIFAS was one of the best scales used. We thought that knowledge about benefits of breastfeeding may be good, but why is the practice of breastfeeding deteriorating? That’s why we thought about examining attitudes.**

A related issue is the possible presence of myths and cultural beliefs related to breastfeeding practice, and how those affect breastfeeding attitudes. These do not seem to have been addressed in the manuscript, though these myths and beliefs are highly prevalent in many societies. The authors should discuss the possible presence or not of breastfeeding myths and cultural beliefs in their study population of Jordan.

**I totally agree with you about the presence of some beliefs about breastfeeding in Jordan, which may be a good idea for a new research paper later on. This may be addressed as a limitation in this study and can be recommendation for future research also.
Added to limitations: “And finally, cultural beliefs about breastfeeding were not addressed as factors that may influence women’s attitude toward breastfeeding in Jordan, which may be the focus of future research”.**

Specific comments:

Abstract.
Second sentence has a misspelling of “determine.”
**It has been corrected.**

Methodology
Medical students were used to interview mothers for this study. What percentage of these were female? There are two intersecting issues. One is that same-sex (female) interviewers may be more likely to obtain truthful answers from female interviewees.

**Medical students who worked on data collection were all females. We have realized this issue and chose females.**

The second is that the interviewees may be also intimidated by perceived better-educated and perceived higher-status medical students, and may provide answers that mothers perceive as the “correct answers” rather than their true attitudes or knowledge.

**This is an inherent bias in a survey (social desirability bias), because of human nature. But we tried to reduce this by using neutrally worded sentences. In addition, anonymous questionnaires should have reduced this.**

Results

Table 3 should indicate that the items were taken from the IIFAS questionnaire with reference cited.

**Title of the table was modified:**

**Table 3. Item scores of mothers’ postnatal attitudes to breastfeeding using the IIFAS (N=301)**

**I think there is no need to reference the IIFAS, since all details were provided in the methods section.**

At the bottom of Table 3, the label should read “Overall mean score on attitudes toward infant feeding = 3.95 + 1.04”.
**Noted. This has been corrected.**

Readers may likely wonder why an attitude score of 3.95 + 1.04 is considered by the authors as a “neutral” attitude. This designation should be reconsidered.
**The cutoff points for attitude to breastfeeding (positive to breastfeeding, neutral, and negative to breastfeeding). This has been frequently used in the literature using the IIFAS, and it has been detailed in the methods section.**

Recommendations

Recommendations are primarily to do more studies on “attitudes.” My question again is related to the issue of breastfeeding “knowledge.” Are more studies needed on breastfeeding knowledge as well as on cultural beliefs and myths related to breastfeeding?

**A recommendation about future research on breastfeeding knowledge was added.**

**Reviewer #7:** The paper is generally well written and structured. Overall, this is a clear, concise, and well-written manuscript. The introduction is relevant. Sufficient information about the previous study findings is presented for readers.

**Thank you. No other comments?**
