# Supplementary material for: Determinants of breastfeeding attitudes of mothers in Jordan: A cross-sectional study
Source: PLoS One. 2023 May 5;18(5):e0285436. doi: 10.1371/journal.pone.0285436 (PMC10162564; doi:10.1371/journal.pone.0285436)
Supplement: Attachment — Submitted filename: Rebuttal letter (revision 3).docx [file pone.0285436.s005.docx]

**PONE-D-22-35675R2**

**Determinants of Breastfeeding Attitudes of Mothers in Jordan: A Cross-sectional Study**

**PLOS ONE**

**Journal Requirements:**

Please review your reference list to ensure that it is complete and correct. If you have cited papers that have been retracted, please include the rationale for doing so in the manuscript text, or remove these references and replace them with relevant current references. Any changes to the reference list should be mentioned in the rebuttal letter that accompanies your revised manuscript. If you need to cite a retracted article, indicate the article’s retracted status in the References list and also include a citation and full reference for the retraction notice.

**Additional Editor Comments:**

Reviewer #4 comment: In table 4, What was the reason for undetermined? explain that in the footnote under the table.

**Response: I have explained the meaning of undetermined in a footnote under the table.**

Make sure you thoroughly address the reviewer’s #6 comments and concerns.


**Reviewers' comments:**

Reviewer's Responses to Questions

**Comments to the Author**

1. If the authors have adequately addressed your comments raised in a previous round of review and you feel that this manuscript is now acceptable for publication, you may indicate that here to bypass the “Comments to the Author” section, enter your conflict of interest statement in the “Confidential to Editor” section, and submit your "Accept" recommendation.

Reviewer #4: All comments have been addressed

Reviewer #5: All comments have been addressed

Reviewer #6: (No Response)

Reviewer #7: All comments have been addressed

**6. Review Comments to the Author**

**Reviewer #4:** (No Response)

**Reviewer #5:** Thank you very much for addressing my comments . The required corrections have been made and the manuscript has been improved scientifically. The authors responded to my comment and provided valuable information which are important for the readers.

**Reviewer #6:**

 Page 2. This paper reports on a study conducted on recently-delivered mothers in a Jordan tertiary-level hospital among whom 75% had university or higher education. The authors should provide data on the average educational level of Jordanian women to provide a better understanding of the generalizability of this study. If the authors intended to help explain the low rate of exclusive breastfeeding in Jordan in order to make policy recommendations, a representative selection of study mothers would have been a better choice. The generalizability of findings needs to be prominently discussed at the end of the paper.

**RESPONSE: The sample was selected from mothers attending this referral teaching hospital in the capital Amman. Many of women who receive service in this hospital are university employees and their families. This explains the high level of education among the participants. These women may not be representative of all women in Jordan. (This has been added to limitations).**

In the introduction, the authors cite literature that higher education is associated with a good or positive attitude toward breastfeeding. The authors should try to explain why the study had measured only an average score of “neutral attitude” toward breastfeeding even though such a high proportion of their study mothers had a high level of education. They should explain in the discussion section that this study has dissimilar results to findings of other studies on the issue of higher education mothers, as they found no association of education with the breastfeeding attitude score.

**RESPONSE: On the other hand, mothers with higher education did not show more positive attitude to breastfeeding. This was inconsistent with results of other studies in the literature, where higher education was significantly associated with more positive attitude to breastfeeding. This may be explained by the fact that mothers with higher education are working or planning to work. For these mothers, bottle-feeding may be considered a more convenient option for infant feeding, since it is less time consuming and can be provided by any care giver. These mothers may also value freedom, and prefer to choose infant feeding method that will not restrict their daily activities.**

Authors should also note that although highest income mothers had a significantly better attitude score as compared to the lowest income mothers, medium low-level income mothers were also significantly different from lowest income. Discuss the implications of this finding.

**RESPONSE: Mothers with higher income level seem to care more about the quality of food they provide for their families, including choosing breastfeeding for their infants. Mothers with lowest income level demonstrated more positive attitude to formula milk feeding. These women cannot afford the price of formula milk, and have no choice but to breastfeed. This may explain why they favor formula milk more, and may perceive it as an ideal nutritious food for their infants.**

The average score of 65 + 7 out of 85 on the IIFAS survey questionnaire was very close to the upper limit of the neutral range 49-69, and could be stated that the tendency was towards a positive attitude.

**RESPONSE: I agree with the reviewer. I restated the description as being close to the upper limit of the neutral range, with tendency toward a positive attitude. I have changed that in the results and in the abstract.**

The authors cite a study by Khasawneb (2020) that found 72% of mothers with a positive attitude toward breastfeeding. The authors should discuss why their findings are so different with only 24% with a positive attitude.
**Response: Khasawneh et al (2020) have overestimated the proportion of mothers with positive attitude to breastfeeding. They offered the choice of only agree or disagree for the IIFAS scale, and agreement with more than half of the items was considered as positive attitude to breastfeeding.**

Page 3. After discussing the importance of exclusive breastfeeding, the authors state that the ‘breastfeeding’ rate in Jordan is 25% and compares this to similar rates reported in other countries. The authors should specify if they are referring to exclusive breastfeeding rates as 25%, or to “any breastfeeding” in children 0-5 months of age.

**Response: It was not exclusive breastfeeding, it was any breastfeeding. It has been clarified in page 3.**

Page 4. In the Introduction, the authors cite two studies conducted in Jordan (2006 and 2018) which reported that positive maternal attitudes toward breastfeeding were associated with exclusive breastfeeding. The authors should provide more details on the socio-demographic-economic characteristics of those two study populations, and also what instruments had been used to measure positive maternal attitude. This would allow the authors to provide a more complete justification of the need for their current study, for their choice of study population, and for their choice of the IIFAS as the attitude measurement instrument.
**Response: The study population in Khasawneh et. al. [****24****] was a randomly selected 344 women from the North of Jordan villages. Using questions adapted from the IIFAS. While Khasawneh et. al. [****27****] interviewed healthy women attending antenatal clinics in three hospitals in the North of Jordan too, using the IIFAS.**

In order for readers to better understand the current research, the paper would benefit from a discussion of the concept of “attitudes”: what exactly is an attitude, types of attitudes, and whether attitudes are predictive of behavior. The same suggestion is made for the concept of “ intentions”, since 95% of the study population had the intention to breastfeed (see Table 2). This discussion should include an explanation of the differences between “attitudes” and “intentions,” as well as the differences between “intentions” and “behaviors”.

**Response:**

**Attitude here is defined as a mental position with regard to a fact or state, and the feeling or emotion toward the fact or state. Attitude is traditionally structured along three dimensions: cognitive, affective, and behavioral. Intention is a prior conscious decision to perform a behavior. Intention can reflect maternal commitment to infant’s health.**

**The breastfeeding behavior is directly determined by the intention to breastfeed. Intention is formed through a combination of attitudes, subjective norm, and perceived behavioural control.**

The data on Table 2 should be included in the sections on results, discussion, conclusions, and recommendations. Based on data reported on Table 2, it appears that the hospital is not carrying out a strong role in promoting positive breastfeeding behaviors. Only 43% of study mothers had breastfed in the first day after birth. Also, only 40% of mothers had received breastfeeding counseling (20% prenatal and 20% postnatal) and 60% had received no counseling. This was in spite of the authors’ statement that the study location hospital was an “accredited Baby Friendly Hospital” since 2018.

**Response:**

**Discussion: Despite the fact that this hospital is a baby friendly hospital, only 43% of mothers initiated breastfeeding in the first day after childbirth, and 60% of mothers did not receive any counselling of advice related to breastfeeding. All this, given that 97.5% of the mothers had the intention to breastfeed. This may be explained by the hospital being a tertiary referral one, receiving a large volume of complicated cases requiring cesarean section, and inducing delay in initiation of breastfeeding. On the other hand, the role of this hospital in promoting positive breastfeeding behavior is suboptimal.**

**Mothers who delivered in this hospital, did not necessarily receive antenatal care in this hospital.**

**In recommendations: In addition, hospitals that are designated as baby friendly, must audit their procedures related to encouraging breastfeeding practice,** **and make sure that related policies are properly implemented.**

**In conclusions: Healthcare professionals at all level of the health care system must play their vital role in promoting breastfeeding, starting with prenatal counselling, and ending with immediate postnatal counselling and supporting mothers to initiate breastfeeding their infants. All this in the attempt to increase to the rates of breastfeeding among women in Jordan.**

In regard to the 43% of breastfeeding on Day 1, the correct WHO definition of early initiation of breastfeeding (EIBF) is “provision of mothers' breast milk to infants within the first hour of birth.” EIBF is the breastfeeding indicator that is most predictive of successful breastfeeding and reduced neonatal and infant morbidity and mortality. If the study questionnaire had included the question on initiation of breastfeeding in the first hour of life, this should be reported in Table 2. Breastfeeding on the first day is not an accepted breastfeeding indicator.

**Response: Data on initiation in the first hour after delivery is not available. It was not the focus of this study.**

Table 4 shows the average score of “attitude toward breastfeeding” (based on the IIFAS) for each category of all the study variables. It is notable that the mean attitude is nearly the same for all categories of all variables. Even for variables such as Working Status of the mother which is mentioned earlier in the paper as a significant factor associated with breastfeeding intentions and practices, the average IIFAS score is exactly the same for both employed and unemployed mothers. The authors should explain why the attitude scores are so very similar for nearly all categories of all variables. One could wonder if the IIFAS is able to detect a difference in attitudes in this study population. If not, the IIFAS could be not really a valid test for this population. On the other hand, if the IIFAS is accurately measuring attitudes toward breastfeeding, then one could conclude that all Jordanian mothers in this population have very similar attitudes toward breastfeeding and in which case, therefore, attitudes are unlikely to be associated with breastfeeding practice. Contrary to findings in this study, in many country studies that could be cited, lower income women are more likely to breastfeed and to exclusively breastfeed for a longer period. That contradiction should be explained.

**Response: Average attitude scores did not differ across different categories, because there were nor real differences in their attitudes.**

**Still, we found attitude to be not associated with actual practice of initiating breastfeeding (and we have discussed this).**

**Our results did indicate that poor women had less positive attitude to breastfeeding. But it was not the focus of this study to analyze the relationship between income level and practice of breastfeeding.**

Statistics

Table 5 with logistic regression results should refer to factors that are “associated” with the attitude score. A cross-sectional observational study cannot infer causality as suggested by the term “predictors.” This issue could extend to the title of the study that uses the term “Determinants.”

**This has been changed.**

Table 5 should include a list of control variables beneath the table, if a step-wise regression was used. Alternatively, the table should include OR and CI results of the entire regression model with all variables included that had had <.10 p value in the bivariate analyses. And next to that, the best-fitting model. Each with r and r2 values reported.

**Response: Results have been revisited and corrected as requested.**

Regarding the data presented on Table 5, there do not seem to be clear results on the variables of “monthly family income” and “willingness to breastfeed exclusively.”

**Response: Results have been revisited, variables added as suggested and finalized.**

On Table 5, the variable on “monthly family income” has 4 levels from low (1) to high (4). Levels 2 and 4 are significant. Level 3 is not significant. These findings cannot be interpreted as only the highest income mothers having significantly better attitudes toward breastfeeding. Rather, the level 2 of income is also significantly different from level 1. (OR 4.72, CI 1.12 – 26.86). The p-level is less important than the CI in regressions.

**Response: interpretation was corrected.**

On Table 5, the variable “willingness to breastfeed exclusively” has confusing confidence intervals. It seems the confidence intervals are inverted for the categories “yes” and “undetermined” and should be reviewed and corrected if necessary.

**Response: 95%CI have been reviewed and corrected.**

Conclusions

Conclusions are not supported by data. The discussion refers to the positive associations of “pregnancy complications,” “delivery complications,” and “intention to breastfeed” with the outcome of attitude score. However, the former variables (pregnancy and delivery complications) have non-significant associations in the logistic regression. The latter variable (intention to breastfeed) is not reported in the logistic regression on Table 5.

**Response: Discussion and Conclusions have been modified in order to match the results of this study.**

Conclusions should be modest and not attempt to go beyond the findings of the study. The study does not show that the attitudes measured are predictive of breastfeeding practice. You will not be able to make conclusions and recommendations about how to improve breastfeeding practice as your data does not provide information on that issue.

**Response: Recommendations have been aligned with results of this study.**

Recommendations

Recommendations should suggest conducting a study that looks at whether the IIFAS is associated with breastfeeding practices in Jordan, controlling for other factors such as hospital breastfeeding routines (early initiation of breastfeeding, rooming-in, etc), newborn characteristics (prematurity, birth weight, birth by cesarean section, etc) and maternal characteristics (educational level, work status, income, etc). This would be a way to test the concept validity of the IIFAS for the Jordanian population of parturient mothers as a predictor of breastfeeding practice. This study would ideally be conducted on a representative population sample with a prospective design so that results can contribute to policy recommendations for the Jordanian population.

**Response:**

**We suggest that future studies look at whether the IIFAS is associated with breastfeeding practices in Jordan, controlling for other factors such as hospital breastfeeding routines (early initiation of breastfeeding, rooming-in, etc), newborn characteristics (prematurity, birth weight, birth by cesarean section, etc) and maternal characteristics (educational level, work status, income, etc).**

**We also recommend that future studies be conducted on a representative population sample with a prospective design so that results can contribute to policy recommendations for the Jordanian population.**
