# Supplementary material for: Determinants of breastfeeding attitudes of mothers in Jordan: A cross-sectional study
Source: PLoS One. 2023 May 5;18(5):e0285436. doi: 10.1371/journal.pone.0285436 (PMC10162564; doi:10.1371/journal.pone.0285436)
Supplement: Attachment — Submitted filename: Response to Reviewers.docx [file pone.0285436.s006.docx]

**PONE-D-22-35675R3**

**Determinants of Breastfeeding Attitudes of Mothers in Jordan: A Cross-sectional Study**

**PLOS ONE**

Additional Editor Comments:

The correct WHO definition of early initiation of breastfeeding (EIBF) is “provision of mothers' breast milk to infants within the first hour of birth.” EIBF is the breastfeeding indicator that is most predictive of successful breastfeeding and reduced neonatal and infant morbidity and mortality.
Since the data on initiation of breastfeeding in the first hour after delivery is not available, so you should add this to the limitations of the study.

**Response: I have added to the limitations about the Lack of data regarding initiation of breastfeeding in the first hour after delivery.**
